# Molecular Antioxidant Functions are Enhanced in Atlantic Bluefin Tuna (*Thunnus Thynnus*, *L*.) Larvae Fed Selenium-Enriched Rotifers *Brachionus Rotundiformis*

**DOI:** 10.3390/antiox12010026

**Published:** 2022-12-23

**Authors:** Pauline Wischhusen, Mónica B. Betancor, Matthew Sprague, Aurelio Ortega, Fernando de la Gándara, Douglas R. Tocher, Gabriel Mourente

**Affiliations:** 1 Institute of Aquaculture, Faculty of Natural Sciences, University of Stirling, Stirling FK9 4LA, Scotland, UK; 2 Planta Experimental de Cultivos Marinos, Instituto Español de Oceanografía (IEO), 30860 Puerto de Mazarrón (Murcia), Spain; 3 Departamento de Biología, Facultad de Ciencias del Mar y Ambientales, Universidad de Cádiz, 11510 Puerto Real, Cádiz, Spain

**Keywords:** bluefin tuna, larvae, Se-yeast, rotifer enrichment, growth performance, gene expression, selenoprotein, antioxidant enzymes

## Abstract

Selenium (Se) is an essential trace element for fish with more than 40 selenoproteins identified, many exhibiting antioxidant functions. This study investigated the effect of dietary Se supplementation on physiological parameters, selenoprotein and antioxidant enzyme gene expression in Atlantic bluefin tuna (ABT, *Thunnus thynnus*) larvae. First-feeding ABT larvae were divided into triplicate groups and fed rotifers *Brachionus rotundiformis* enriched with five different levels of Se (0, 3, 10, 30, and 100 µg Se·L^−1^) until 14 days after hatching. Both rotifers and ABT larvae effectively accumulated Se achieving maximum levels in the Se100 treatment (30.05 μg Se·g^−1^ and 194 ± 38 μg Se·g^−1^ dry mass, respectively). Larvae showed highest total length when fed Se3 rotifers, whereas flexion index was highest in larvae fed Se10. Selenium supplementation increased the gene expression of selenoproteins *gpx1*, *msrb1, trxr2*, *selenom*, *selenop*, and *selenoe* compared to the non-supplemented control (Se0), but only marginal differences were detected between supplementation levels. In contrast, expression of the antioxidant enzymes *cat* and *sod1* were lowest in larvae fed Se100. To conclude, non-Se-enriched rotifers may be suboptimal for first feeding ABT larvae, which showed improved selenoprotein and antioxidant gene expression when fed a diet containing 4.42 μg Se·g^−1^ dry mass.

## 1. Introduction

Atlantic bluefin tuna (ABT, *Thunnus thynnus,* L.) is a high-value species on the market. In recent years, ABT juveniles have been captured from the wild and fattened using bait fish in so-called sea ranches [1]. Recently the production cycle of ABT has been closed, however low survival at larval stages dictates this is still far from commercial production [1,2]. As in other bluefin tuna species, ABT has a very sensitive larval stage characterized by several problematic issues including size variation, low swim bladder inflation rates and skeletal anomalies [3,4]. Improved knowledge of the nutritional requirements of larvae could be one solution to boost ABT production where no standard hatchery feeding protocol exists. Although some studies have been performed on different aspects of ABT nutrition [5,6,7,8] there is limited information regarding requirements and functionality for many micronutrients, especially at the larval stage.

Micromineral selenium (Se) is essential for health, normal growth and development in fish [9]. Although the Se requirement of ABT is currently unknown, wild tuna presents high levels of body Se that may be a response to counteract toxic effects of heavy metals prone to accumulate in this top predator species [10]. Fish obtain Se mainly from the diet with levels to satisfy requirements ranging between 0.15 and 1.85 µg·g^−1^, however, toxic effects can be observed at marginally higher levels [11]. Recent studies suggested that rotifers, which are also used as starter feeds for ABT, might not cover the nutritional requirements of marine finfish species [10,12]. As Se requirements are known to increase in response to oxidative stress, Se supplementation may prove especially beneficial in this fast-growing species at early life stages, which are especially sensitive to changing environmental conditions [13,14,15].

Oxidative stress derives from an imbalance between the production of reactive oxygen species (ROS) and the cellular antioxidant defence system [16,17]. While ROS are indispensable for cell signalling, excessive production leads to the oxidation of cellular compounds such as proteins and lipids. Cellular antioxidants can prevent, lower or reverse the damage caused by ROS. The antioxidant system is composed of nutritional compounds such as vitamins C and E that scavenge ROS, but also consists of several key enzymes including catalase (CAT), superoxide dismutase (SOD) and glutathione peroxidases (GPx) that catalyse the decomposition of hydrogen and lipid peroxides [18]. Seven GPx enzymes, that use glutathione as a substrate to reduce peroxides, have been described in fish out of which five were identified as selenoproteins [19]. Selenoproteins have selenocysteine incorporated at the active centre via a process of co-translational insertion in response to specific UGA codons and the presence of a cis-acting specific selenocysteine insertion sequence SECIS element [20]. Fish have one of the largest selenoproteoms identified among vertebrates [19]. Besides GPx, other selenoproteins are well characterized for their role in the antioxidant system including methionine sulfoxide reductase with its ability to recover oxidized methionine preventing the disruption of proteins or thioredoxin reductase with its ability to reduce oxidized thioredoxins [21,22,23]. Selenoprotein P was the first selenoprotein described to contain more than one selenocysteine. It acts as a seleno-transporter and its expression is often used to determine Se requirements [24]. Other selenoproteins are less well characterized to date, especially in fish, although many of them were shown to have antioxidant properties [25].

In the above context, the present study aimed to investigate the dose-dependent effect of dietary Se level on growth performance, the expression of selenoproteins and antioxidant enzymes, and oxidative status in ABT larvae.

## 2. Materials and Methods

### 2.1. Ethical Statement

This feeding trial was performed under the regulations on the protection of animals used for scientific purposes as defined by the European Directive 2010/63/EU (European Parliament and Council, 22 September 2010) and the Spanish legislation RD 53/2013 (BOE 8 February 2013). Additionally, ethical approval was granted by the Animal Welfare and Ethical Review Board (AWERB) of the University of Stirling, UK (ID TunaSe8456).

### 2.2. Atlantic Bluefin Tuna Larvae Rearing Conditions

The ABT eggs were obtained from naturally spawning ABT broodstock fish cultivated in floating net cages off the Cartagena coast, El Gorguel, SE Spain in July 2018. Collected eggs were transported under provision of oxygen to the Spanish Institute of Oceanography (IEO) Planta Experimental de Cultivos Marinos (Puerto de Mazarrón, Murcia, Spain) and placed in 100 L flow-through tanks equipped with oxygenized and sterilized seawater. To separate buoyant (viable) from non-buoyant (non-viable) eggs aeration and water flow were stopped after 1 h. All viable eggs were washed, disinfected (25 ppm iodine solution, Germiod, CENAVISA, SA, Reus, Spain) and counted. Fertilized eggs were kept in 1400 L cylindrical tanks under standardized conditions (24 °C water temperature, 37‰ salinity, 6.5 mg·L^−1^ dissolved oxygen and continuous photoperiod of 1000 lux) at a density of 8.5 eggs·L^−1^. Larvae hatched approximately 32 h after fertilization, with a hatching rate of almost 90%, and were fed the Se-enriched rotifers *Brachionus rotundiformis* from 2 days after hatching (dah). To create green-water, a mixture of the microalgae *Isochrysis* sp. (T-Iso) and *Chlorella* (V12 DHA-enriched, Pacific Trading Co., Fukuoka, Japan) were added to tanks at a density of 2–3 × 10^5^ cells mL^−1^. During larvae feeding, photoperiod was maintained at 14 h light/10 h dark (light intensity about 1000 lux, from 7:30 h to 20:30 h), temperature ranged between 24–26 °C and daily water renewal was 100–200% tank volume·day^−1^. All incoming seawater was filtered at 10 µm size and UV sterilized. Larvae sinking (mainly at night) was prevented through the creation of an upwelling current which also helped to stabilize oxygen levels [5,6,26].

### 2.3. Dietary Treatments

Rotifer (*Brachionus rotundiformis*) grown on Algamac 3050 (Pacific Trading LTD, Kent, England) was supplemented with different levels of seleno-yeast, SelPlex^®^, Alltech (Meath, Ireland) and used as larvae feed. Rotifer enrichment took place for 18 h in culture medium supplemented with SelPlex^®^ at the following doses ranging between non-supplemented to potentially excessive: 0.0 mg/10^6^ rotifers (0 μg Se·L^−1^, Se0), 3 mg/10^6^ rotifers (3 μg Se·L^−1^, Se3), 10 mg/10^6^ rotifers (10 μg Se·L^−1^, Se10), 30 mg/10^6^ rotifers (30 μg Se·L^−1^, Se30) and 100 mg/10^6^ rotifers (100 μg Se·L^−1^, Se100). Selenium levels in rotifers were quantified as described in paragraph 2.6.1. The treatments and final analysed Se levels in freeze dried rotifers are summarized in Table 1.

Each dietary treatment consisted of three triplicate 1500 L cylindro-conical tanks equipped with ABT larvae at a fish density of 10 larvae·L^−1^. Manual feeding ensured a constant prey density of 5 rotifers·mL^−1^ throughout the trial.

### 2.4. Sample Collection of Rotifers and ABT Larvae: Sampling for Growth Performance, Biochemical and Molecular Analysis

At sampling, individual fish were photographed and lengths, weight and developmental stage were determined on twenty-five anaesthetized fish (0.02% 2-phenoxyethanol, Sigma, Spain) per triplicate treatment. To determine the developmental stage larvae that achieved full flexion of the notochord by the end of the feeding trial (14 dah) were counted. Whole larvae were dried at 110 °C for 24 h and cooled in vacuo for 1 h before weighing in a precision balance to determine individual dry mass.

For each treatment three subsets of triplicate samples were collected to either determine dry mass (20 individuals), perform biochemical analysis (50 individuals) or molecular analysis (50 individuals). Larvae dedicated for molecular analysis were placed in 1.5 mL of RNAlater^®^ (Sigma, Madrid, Spain) before all samples were snap-frozen in liquid nitrogen and stored at −80 °C until further analysis.

### 2.5. Larvae Biometry and Survival

At 1, 2, 3, 6, 8, 12 and 14 dah, 25 larvae per tank (replicate) were sampled for total length. Therefore, images were taken using a camera (Olympus SC20, OLYMPUS, Münster, Germany) connected to a microscope (Olympus SZ61-TR, Leica, Hamburg, Germany). Then, Image Pro 6.2 (Media cybernetics; Buckinghamshire, UK) software was used to determine total length. Survival (%) represents the difference in individual live larvae at the beginning and at the end of the trial.

### 2.6. Biochemical Analysis

#### 2.6.1. Selenium Analysis

Selenium was measured in 40–80 mg of dried rotifers and ABT larvae. The samples were measured in triplicate by digestion in 5 mL of 65% nitric acid using a microwave digester (Mars6, CEM, Charlotte, NC, USA) with the following settings: 21 °C to 190 °C for 10 min at 800 W, then 190 °C for 20 min at 800 W and finally 30 min cooling. The digested samples were two times diluted in distilled water and 0.2 mL methanol, and total Se was measured by Inductively Coupled Plasma Mass Spectrometry (Thermo Scientific, XSeries2 ICP-MS, Waltham, MA, USA) using the carrier gases argon and hydrogen.

#### 2.6.2. Total Lipid, Fatty Acid and TBARS Analysis

Total lipids of ABT larvae were extracted from pooled samples according to the method of Folch et al. (1957) [27]. Approximately 50 mg dried ABT larvae were homogenized and processed as described in detail previously [6] before the lipid content was determined gravimetrically. 

Fatty acid methyl esters (FAME) were analysed from the extracted total lipids according to the method of Christie [28,29]. Gas-liquid chromatography (Agilent Technologies 7890B GC System) equipped with a 30 m × 0.32 mm i.d. fused silica capillary column (SUPELCOWAX^TM^-10, Supelco Inc., Bellefonte, PA, USA) was used to separate and quantify FAME as described previously in Betancor et al. (2017) [6].

Thiobarbituric acid reactive substances (TBARS), formed as a by-product of lipid peroxidation, were measured in total lipid extracts. Briefly, 0.1 mg of total lipids were evaporated to dryness under oxygen-free nitrogen. Next, 0.5 mL of 1% thiobarbituric acid, 0.5 mL of 10% trichloracetic acid and 50 µL of 0.02% BHT in ethanol were added and the samples placed in a 100 °C water bath for 20 min. Finally, absorbance was measured at 532 nm against a reagent blank.

### 2.7. Molecular Analysis

#### 2.7.1. Tissue RNA Extraction and cDNA Synthesis

Total RNA extraction was performed on duplicate pools of 15 whole ABT larvae per tank (to provide 6 samples per dietary treatment) using 1 mL TriReagent^®^ (Sigma-Aldrich) extraction buffer. Quantity and quality of RNA was measured using a Nanodrop ND-1000 (Labtech Int., East Sussex, UK) and additionally by electrophoresis running 200 ng of total RNA on a 1% agarose gel. Reverse transcription was performed with random primers on 2 µg total RNA using the High Capacity cDNA Reverse Transcription kit (Applied Biosystems, REF4368813, Warrington, UK) according to the manufacturers protocol.

#### 2.7.2. Primer Preparation and qPCR

The primer sequences used in the quantitative real-time PCR (qPCR) are given in Table 2.

Primers for *gpx1*, *gpx4*, *cat*, *sod1*, *ef1a* and *bactin* were already available [5]. The other primers were designed on gene sequences available in close related tuna species and on the sequence read archive (SRA) of ABT (SRX22557558, SRX2766917) by identifying and assembling the sequences. The efficiency of primers was evaluated on a serial dilution of ABT cDNA to verify it was >84% for all primer pairs.

The qPCR was performed in a qTower^3^ G real-time PCR Thermal Cycler (Analytic Jena GmbH, Jena, Germany) under the following conditions: 50 °C for 2 min, 95 °C for 10 min, then 34 cycles of 95 °C for 15 s, 60 °C for 30 s and 72 °C for 30 s, then 6 s at the annealing Tm. The total reaction volume of 10 µL compromised 5 µL Luminaris Color HiGreen qPCR Master Mix (Thermo Scientific, Hemel Hempsted, UK), 0.5 µL primer pairs at 10 pmol concentration, 1.5 µL deionised water and 2.5 µL cDNA (1/20 dilution). The housekeeping genes were measured in the same reaction volume but diluted at 1/200. A non-template control containing no cDNA was measured alongside each gene and the melting curves were systematically screened. 

The relative expression levels of target genes were calculated as geometric means of the housekeeping genes *ef1a* and *bactin* using the ΔΔCT method on the average of the control treatment Se0 [30].

### 2.8. Statistical Analysis

Results are given as mean ± standard deviation. Data were analysed using statistical software R (R Development Core Team, 2021, Vienna, Austria). Normality and homogeneity were verified prior to model building resulting in rank transformation of gene expression data. Dietary treatments were analysed by one-way ANOVA followed by a Tukey’s HSD post hoc test and considered significantly different at *p* < 0.05.

## 3. Results

### 3.1. Performance of ABT Larvae during the Feeding Trial

ABT larvae fed Se3-rotifers showed the numerically highest survival, significantly better than those fed the Se10-rotifers (Table 3). Total length of larvae fed the non-supplemented rotifers was lower compared to larvae fed Se3- or Se30-rotifers with intermediate values for larvae fed the Se10 and Se100 treatments (Table 3). Similarly, ABT larvae fed Se0-rotifers had a lower dry mass than those fed Se30-rotifers. The flexion index was increased in larvae fed all Se-enriched treatments, but was highest in ABT larvae fed Se10- and Se30-rotifers.

### 3.2. Whole Body Se Content of ABT Larvae

**Figure 1:**
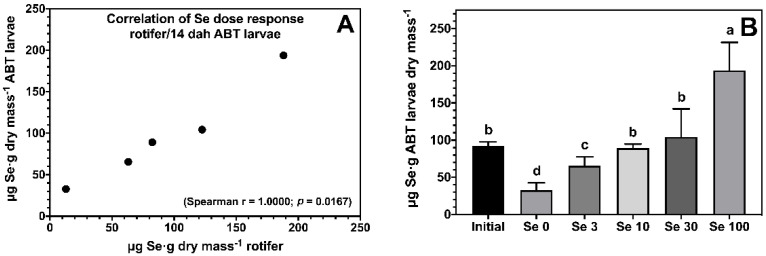
Dose response correlation between selenium levels in rotifers *Brachionus rotundiformis* grown on Algamac 3050 Bio Marine^®^ and enriched with 5 graded selenium levels (0, 3, 10, 30, and 100 mg of Se-yeast per 10^6^ rotifers) and ABT larvae fed the rotifers from mouth-opening until 14 days post-hatching (**A**). Total body selenium levels (*n* = 3; mean ± SD) measured in ABT larvae at the end of the feeding trial (**B**). Bars not sharing a common superscript letter indicate significant differences (*p* < 0.05) between groups according to one-way ANOVA followed by Tukey’s HSD.

The enrichment of rotifers effectively increased body Se levels of ABT larvae, and showed a strong dose-dependent correlation (Figure 1A). All ABT larvae fed rotifers enriched with Se showed significantly higher body Se levels compared to the negative control treatment Se0 (Figure 1B). ABT larvae fed the Se100 treatment accumulated 194 ± 38 μg Se·g^−1^ dry mass followed by larvae fed Se10 and Se30, with larvae fed Se3 showing the lowest body Se levels within the supplemented treatments.

### 3.3. Total Lipid Fatty Acid Composition and TBARS Content of ABT Larvae

Feeding Se-enriched rotifers had no major impact on the fatty acid composition of ABT larvae other than some small, likely not biologically significant, variations in proportions of quantitively minor fatty acids, 18:3n − 6, 20:3n − 6 and 20:4n − 3 (Table 4). Similarly, the TBARS concentration as a measure of lipid peroxidation was not significant different between the groups (Table 4).

### 3.4. Gene Expression

#### 3.4.1. Expression of Selenoproteins in ABT Larvae

**Figure 2:**
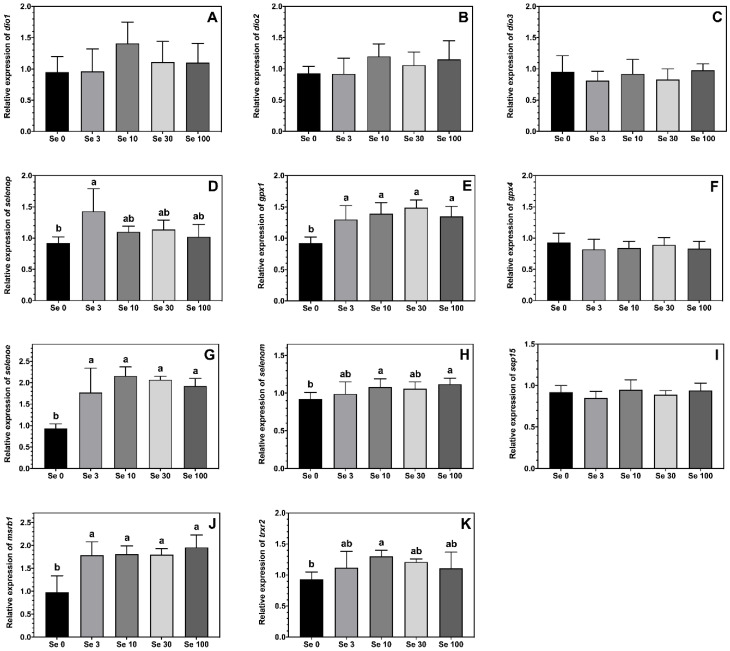
Expression levels (means ± SD, *n* = 6) of selenoproteins iodothyronine deiodinase 1, *dio1* (**A**); iodothyronine deiodinase 2, *dio2* (**B**); iodothyronine deiodinase 3, *dio3* (**C**); selenoprotein p, *selenop* (**D**); glutathione peroxidase 1, *gpx1* (**E**); glutathione peroxidase 4, *gpx4* (**F**); selenoprotein E, *selenoe* (**G**); selenoprotein M, *selenom* (**H**); 15-kda selenoprotein, *sep15* (**I**); methionine sulfoxide reductase 1, *msrb1* (**J**) and thioredoxin reductase 2, *trxr2* (**K**) measured by real-time PCR. Data were normalized to a geometric mean of *ef1a* and *bactin* and are presented as fold-changes of mRNA abundance compared with Se0. One-way ANOVA on ranks followed by Tukey’s HSD postdoc test was used to detect significant differences as displayed through uncommon superscript letters.

The expression levels of the selenoproteins *gpx1*, *msrb1* and *selenoe* were higher in ABT larvae fed all the Se-enriched rotifers compared to the non-enriched Se0 treatment (Figure 2E,G,J). A similar pattern was observed for the expression levels of *selenop*, *trxr2* and *selenom*, although the differences were only statistically significant between larvae fed Se0 vs. Se3 for *selenop*, Se0 vs. Se10 for *trxr2*, and Se0 vs. both Se10 and Se100 for *selenom*, with fish from other treatments showing intermediate values (Figure 2D,H,K). The feeding of Se-enriched rotifers had no significant effect on the expression levels of *gpx4, sep15, dio1, dio2* and *dio3* in the ABT larvae (Figure 2A–C,F,I).

#### 3.4.2. Expression of Other Antioxidant Defence Genes in ABT Larvae

**Figure 3:**
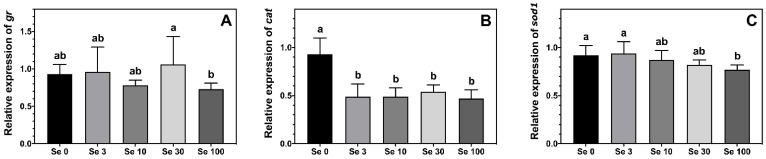
Expression levels (means ± SD, *n* = 6) of antioxidant enzymes gluthatione reductase, *gr* (**A**); catalase, *cat* (**B**) and superoxide dismutase 1, *sod1* (**C**) measured by real-time PCR. Data were normalized to a geometric mean of *ef1a* and *bactin* and are presented as fold-changes of mRNA abundance compared with Se0. One-way ANOVA on ranks followed by Tukey’s HSD postdoc test was used to detect significant differences as displayed through uncommon superscript letters.

The expression level of *gr* was highest in ABT larvae fed Se30-rotifers compared to those fed the Se100 treatment, while larvae fed rotifers enriched with 0–10 mg doses of Se-yeast displayed an intermediate level of expression (Figure 3A). The expression of the antioxidant enzyme *cat* was lower in ABT larvae fed all the Se-enriched rotifers compared to larvae fed the control supplemented rotifers (Figure 3B). Additionally, the expression of *sod1* in ABT larvae reflected the Se-enrichment in the rotifers (Figure 3C). In this sense, the highest *sod1* expression was measured in the two lowest Se treatments Se0 and Se3, while the lowest expression was observed in larvae fed the Se100 rotifers, the highest Se-enrichment. On the other hand, ABT larvae fed Se10- and Se30-rotifers, the medium enrichment treatments, displayed intermediate values for *sod1* expression.

## 4. Discussion

The Se requirement for ABT is currently unknown. In a dose-dependent feeding trial this study could show for the first time that optimized Se feeding has the potential to improve ABT larval performance under aquaculture conditions. The further molecular analysis revealed that these changes were accompanied with modifications in the antioxidant metabolism of the fish.

Selenium enrichment had no significant effect on the survival of ABT larvae in the present trial. This was similar to a previous study in Senegalese sole (*Solea Senegalensis*) larvae where no differences in survival were detected by enriching rotifers with 3 mg Se-yeast per million [31]. The higher survival previously described in cod (*Gadus morhua*) larvae fed Se-supplemented rotifers might be a result of simultaneous co-enrichment with iodone, as enrichment with Se alone had no significant effect on survival of red sea bream (*Pagrus major*) larvae [32,33]. In the present study, the lower survival observed in ABT larvae fed rotifers enriched with 10 mg compared to 3 mg Se-yeast per million rotifers does not appear to be a result of excessive Se supplementation as decreased survival was not observed at the highest Se enrichment level, in line with previous observations in red drum (*Sciaenops Ocellatus*) larvae where adverse effects on growth were only reported at Se supplementation levels above 50 mg/L [34].

In the present trial, growth, in terms of total body length and weight, was lowest in ABT larvae fed unenriched rotifers, however, a significant difference was only detected in larvae fed the rotifers enriched with 3 and 30 mg Se-yeast. The improved growth with Se supplementation observed in this study might be related to accelerated development as the flexion index was significantly higher in larvae fed all Se-enriched treatments compared to the non-supplemented control. Indeed, an earlier study in red sea bream larvae showed similar effects in response to dietary Se supplementation [33]. Interestingly, a study on Senegalese sole reported that Se enrichment modified the activity of the thyroid hormone T_4_ during critical phases of larval development [31,35]. The iodothyronine deiodinases (DIOs) are selenoproteins, therefore, their expression can be enhanced by higher levels of tissue Se. In the present study, whole-body Se content in ABT larvae increased in a dose-dependent manner with Se supplementation levels. Nonetheless, the expression of selenoproteins follows a principle of hierarchy and, in comparison to other selenoproteins, the expression of DIOs, where absence can be lethal, is known to be generally stable and will rather decrease with pronounced Se deficiency [36]. This could explain why no significant differences were observed between treatments for the expression of DIOs, but also with some of the other selenoproteins in the present study. Indeed, the Se content of the control non-supplemented rotifers, with 0.10 µg Se g^−1^ rotifers, in this study, is lower than the Se requirements reported for other fish species, which range between 0.15 and 1.85 µg Se g^−1^ diet in comparison to the lowest supplementation level which provided 4.42 µg Se g^−1^ rotifers which is similar to the optimal dietary Se level reported for red drum larvae [11,34]. Consistent with this, the expression of *selenop,* which is often used as an indicator for Se requirements, was higher in ABT larvae fed the lowest supplementation treatment (Se3) compared to the non-supplemented control, but was not affected by higher enrichment levels in this study. Similarly, the expression of seleno-dependent GPx is known to increase according to Se level making it an indicator for optimal Se status of fish larvae [37]. In the present study, the expression of *gpx1*, but not *gpx4*, was elevated by Se supplementation but, indeed, *gpx4* ranks higher in selenoprotein hierarchy [36]. Glutathione peroxidases are key enzymes for the reduction of lipid peroxides using glutathione as a substrate [38]. The current data show that other selenoproteins with an active role in the antioxidant system were also upregulated in ABT larvae fed the Se-enriched rotifers. These included *msrb1* with its ability to recover oxidized methionine thus preventing the disruption of proteins, and *trxr2* with its ability to reduce oxidized thioredoxins [21,22,23]. In contrast, the thioredoxin-like protein *sep15*, which is involved in antioxidant protection of the thyroid gland was not affected by Se supplementation in the present trial [39]. Nevertheless, in this study, the total Se requirements of ABT larvae might be masked by the mobilization of parentally transferred Se as indicated by the higher total body Se levels detected in the initial control samples compared to larvae at final sampling. The Se requirements in ABT broodstock are currently unknown, but as previously demonstrated in rainbow trout (*Oncorhynchus mykiss*) parentally transferred Se can induce modifications in the antioxidant metabolism of first feeding fry [40].

Contrary to our expectations, the expression levels of selenoproteins in ABT larvae showed few differences among the Se-enriched groups, which indicated that the lowest supplementation level might have already provided sufficient Se to support adequate selenoprotein production in this study. Surplus Se may not have been translated to selenoproteins, but rather stored as other forms. In the blood of wild tuna, more than 98% of total Se was identified as selenoneine, an organic Se analogue of ergothionine with strong antioxidant properties [41]. However, the question is if similar findings could be expected in tuna originating from closed-cycled aquaculture. It is currently unknown whether selenoneine originates from the diet and accumulates in the tissue of tuna as a top predator species or if it is synthesised. Selenoneine was detected in mackerels, a common prey for tuna, but high levels of selenoneine were also detected in beluga whales, but not their prey [42]. Selenoneine is synthesised by similar enzymes as ergothionine, a process so far only described in a limited number of bacterial and fungal species but never in vertebrates [43]. Nevertheless, selenoneine could be a product of specific microorganisms present in the gut microbiome of tuna species and, indeed, could represent a potent antioxidant besides selenocysteine, which is the active Se form in selenoproteins, and should therefore be analysed in future studies.

It has been suggested that the vital role of Se in the antioxidant system can also help to prevent oxidative stress resulting from the provision of live feeds which are often specifically enriched with high levels of n − 3 long-chain polyunsaturated fatty acids that can promote pro-oxidant conditions. Supplementation with Se (and iodine) was reported in a previous study to lead to modifications in the fatty acid profile of cod larvae [32]. However, in the present study the fatty acid profiles of ABT larvae displayed no differences between larvae fed rotifers supplemented with Se, with similar results previously observed in red sea bream [33]. Additionally, the levels of TBARS, which are by-products of lipid oxidation, were not significantly different between ABT larvae fed the different treatments giving no indication of elevated oxidative stress in any of the present treatments. However, the fatty acid profile of fish larvae predominantly reflects the fatty acid profile of the diet, i.e., the rotifers [44]. Live feeds that do not bring a standardised nutritional profile add a factor of uncertainty when comparing studies [12,32]. This is especially true for investigations on microminerals, which can substantially deviate between different batches of live feeds. In the present study, another indicator that indeed supports changes in the cellular redox state of ABT larvae by Se supplementation, especially at higher levels, is the reduced gene expression of the two antioxidant enzymes *sod1* and *cat*. While these enzymes are not selenoproteins and therefore not directly regulated by Se supply, they could be regulated indirectly by the impact of Se on redox status through redox sensitive transcription factors [45,46]. This shift in the transcription of antioxidant genes should be further investigated in future studies focusing on the effects of post-transcriptional enzyme regulation and the glutathione metabolism.

## 5. Conclusions

The present study showed that rotifers without Se enrichment might provide levels of Se that are suboptimal for ABT larvae at first feeding. Se supplementation improved growth performance as well as selenoprotein gene expression. Improved oxidative status in ABT fed Se-enriched rotifers was indicated by lower antioxidant enzyme gene expression, albeit not supported by measures of lipid oxidation. The lack of significant differences in selenoprotein gene expression between larvae fed higher levels of Se supplementation suggested that a dietary Se level of 4.42 μg·g^−1^ DM may be sufficient to satisfy Se requirements in ABT larvae.

## Figures and Tables

**Table 1:** Supplementation levels and analysed selenium concentrations in rotifer *Brachionus rotundiformis* enriched in culture medium for 18 h with different doses of seleno-yeast (SelPlex^®^) and used as starter feeds for ABT larvae in the feeding trial.

Treatment	Supplementation Level [μg Se·L^−1^]	Analysed Level [μg Se·g^−1^ DM]
Se0	0	0.10
Se3	3	4.42
Se10	10	8.95
Se30	30	12.49
Se100	100	30.05

**Table 2:** Primer sequences used to assay mRNA levels by real-time PCR.

Gene	Forward Primer	Reverse Primer	Amplification Size	Tm °C	Gene Accession Number
*selenop*	TTTCAGTAAAAGGATTGGGGCAGT	CCACCTCCCCTATCTTCCAGG	159	60	XM_042422385
*gpx1*	TGGAGAAAGTGGATGTGAACGG	GTGCTGTGGAAGCTGTATGATGG	309	55	XM_042407639
*gpx4*	TGGGGAATAGCATCAAGTGG	CGAGAAAGGAGGGAAACAGG	206	55	XM_042417456
*msrb1*	AAGTTCTTCGGGGGAGAGGT	CCGTACCTTGTATGCCCCAG	192	60	XM_042392867
*trxr2*	GCAACGAACACAAGGACACC	TTCCCCGTCTCGTTGTTGAG	115	60	XM_042421509
*selenoe*	AAGAGGTCCCAGAAAGGGGA	GTCCTCAGAATGGTGCCTGG	177	60	XM_042422277
*selenom*	GGATCGGATCGCCTTGTCTG	GTGGAGGGCTTGTACTCTGG	168	60	XM_042420960
*sep15*	TTGTCAGGAGTGACAAGCCG	TCGGCGATGTTCCCGTTATC	106	60	XM_042427572
*dio1*	TTGCACCTGACCACCGTTTA	CGGACAGCCTTTCCTCCAAA	179	60	XM_042417653
*dio2*	GAAAGTCGGGAGCACTCCAT	GTCACGAGCAGATCCATCCC	167	60	XM_042389463
*dio3*	CGCAGTCGCATCCTCGATTA	CGGTGCTTGGGAATCTGGTA	218	60	XM_042388824
*gr*	TGTAGCTCATGTGAGGATCACC	AGAGGCAGGGAGCTCTAGTC	200	60	XM_042419753
*cat*	ATGGTGTGGGACTTCTGGAG	ATGAAACGGTAGCCATCAGG	95	60	XM_042411457
*sod1*	TCCCAGATCACCTACATGCC	CTGCGGAGAGTTGCTTGATC	182	59	XM_042402399
*ef1a*	CCCCTGGACACAGAGACTTC	GCCGTTCTTGGAGATACCAG	119	60	XM_042435016
*bactin*	ACCCACACAGTGCCCATCTA	TCACGCACGATTTCCCTCT	155	61	XM_042393876

*selenop*, selenoprotein P; *gpx*, glutathione peroxidase; *msrb*, methionine sulfoxide reductase; *trxr*, thioredoxin reductase; *selenoe*, selenoprotein E; *selenom*, selenoprotein M; *sep15*, 15-kda selenoprotein; *dio*, iodothyronine deiodinase; *gr*, glutathione reductase; *cat*, catalse; *sod1*, superoxide dismutase; *ef1a,* eukaryotic translation elongation factor 1α; *bactin*, β-actin.

**Table 3:** Growth and survival of ABT larvae at 14 days after hatch fed rotifers *Brachionus rotundiformis* grown on Algamac 3050 Bio Marine^®^ and enriched with graded levels of selenium (0, 3, 10, 30, and 100 mg of Se-yeast per 10^6^ rotifers).

	Se0	Se3	Se10	Se30	Se100
Survival [%]	6.5 ± 0.9 ^ab^	10.1 ± 1.5 ^a^	5.9 ± 1.1 ^b^	6.8 ± 0.5 ^ab^	7.5 ± 2.6 ^ab^
T. length [mm]	6.6 ± 0.6 ^b^	7.0 ± 0.6 ^a^	6.8 ± 0.7 ^ab^	7.0 ± 0.5 ^a^	6.7 ± 0.5 ^ab^
Dry mass [mg]	0.6 ± 0.1 ^b^	0.7 ± 0.1 ^ab^	0.7 ± 0.1 ^ab^	0.7 ± 0.1 ^a^	0.6 ± 0.1 ^ab^
Flexion Index	69 ± 5 ^c^	76 ± 14 ^b^	80 ± 8 ^a^	77 ± 6 ^a^	74 ± 3 ^b^

Results are means ± SD, 3 replicates/tanks per treatment (total length: *n* = 75; dry mass and flexion index: *n* = 30; survival: *n* = 3). Values within rows not sharing a common superscript letter are significantly different (*p* < 0.05) according to one-way ANOVA followed by Tukey’s HSD.

**Table 4:** Total lipid (mg·g dw^−^^1^), fatty acid composition (% of total fatty acids) and TBARS content (nmol·g lipid^−^^1^) of ABT larvae fed rotifers *Brachionus rotundiformis* enriched with Algamac 3050 Bio Marine^®^ and graded levels of selenium (0, 3, 10, 30, and 100 mg of Se-yeast per 10^6^ rotifers) for 13 days.

	Se0	Se3	Se10	Se30	Se100	*p*-Value
**Total lipid**	12.1 ± 0.9	12.1 ± 0.3	11.4 ± 0.8	12.1 ± 0.6	12.3 ± 0.4	0.57
						
**Fatty acid**						
16:0	23.56 ± 1.47	22.77 ± 0.73	22.2 ± 0.4	22.4 ± 0.2	22.8 ± 0.8	0.38
18:0	11.95 ± 0.95	12.0 ± 0.5	11.4 ± 0.4	11.6 ± 0.1	11.7 ± 0.2	0.64
**Total saturated ^§^**	39.3 ± 2.4	38.5 ± 1.1	37.4 ± 0.8	37.8 ± 0.3	38.4 ± 1.2	0.51
						
18:1n − 9	4.45 ± 0.30	4.11 ± 0.51	3.77 ± 0.03	3.86 ± 0.16	4.86 ± 0.89	0.11
**Total monounsaturated ^#^**	11.5 ± 0.6	11.1 ± 0.3	11.1 ± 0.0	11.0 ± 0.2	12.4 ± 1.6	0.24
						
18:2n − 6	9.91 ± 0.54	9.01 ± 0.57	9.32 ± 0.04	9.57 ± 0.40	10.1 ± 0.8	0.20
18:3n − 6	0.11 ± 0.00 ^b^	0.12 ± 0.01 ^ab^	0.12 ± 0.00 ^a^	0.11 ± 0.00 ^ab^	0.11 ± 0.00 ^b^	**0.02**
20:3n − 6	0.36 ± 0.01 ^ab^	0.34 ± 0.01 ^b^	0.38 ± 0.00 ^a^	0.37 ± 0.01 ^ab^	0.37 ± 0.01 ^ab^	**0.02**
20:4n − 6	2.22 ± 0.17	2.58 ± 0.2	2.53 ± 0.08	2.49 ± 0.13	2.34 ± 0.29	0.21
22:5n − 6	4.51 ± 0.21	5.3 ± 0.16	5.28 ± 0.12	5.02 ± 0.39	4.26 ± 0.95	0.09
**Total n − 6 PUFA ^+^**	18.4 ± 0.5	18.5 ± 0.2	18.8 ± 0.2	18.8 ± 0.2	18.4 ± 0.4	0.36
						
18:3n − 3	1.61 ± 0.11	1.52 ± 0.16	1.61 ± 0.03	1.58 ± 0.05	1.77 ± 0.25	0.37
20:4n − 3	0.37 ± 0.01 ^c^	0.36 ± 0.02 ^bc^	0.41 ± 0.01 ^a^	0.38 ± 0.01 ^abc^	0.40 ± 0.01 ^ab^	**<0.01**
20:5n − 3	3.2 ± 0.27	3.02 ± 0.2	3.25 ± 0.14	3.26 ± 0.13	3.36 ± 0.19	0.37
22:6n − 3	20.62 ± 1.3	22.1 ± 0.9	22.2 ± 0.5	22.0 ± 0.3	19.9 ± 2.3	0.19
**Total n − 3 PUFA ^&^**	28.9 ± 2.0	29.9 ± 1.1	30.7 ± 0.8	30.4 ± 0.1	28.8 ± 1.8	0.35
						
**Total PUFA**	48.0 ± 2.4	49.0 ± 1.2	50.2 ± 0.7	49.9 ± 0.3	47.9 ± 2.1	0.33
						
**n − 3/n − 6**	1.57 ± 0.07	1.61 ± 0.06	1.63 ± 0.05	1.62 ± 0.02	1.56 ± 0.07	0.52
**EPA ± DHA [g/100 g]**	1.16 ± 0.14	1.29 ± 0.09	1.30 ± 0.14	1.31 ± 0.09	1.24 ± 0.15	0.56
						
**TBARS**	996 ± 263	840 ± 161	1053 ± 190	1011 ± 250	957 ± 198	0.82

Results are means ± SD (*n* = 3). Mean values within a row with different superscript letters were significantly different according to one-way ANOVA followed by Tukey’s HSD post hoc test. ^§^ Totals include 14:0, 15:0, 20:0, 22:0, 23:0 and 24:0; ^#^ Totals include 16:1n − 9, 16:1n − 7, 17:1, 18:1, 18:1n − 7, 20:1n − 11, 20:1n −9, 20:1n − 7, 22:1n − 11, 22:1n − 9 and 24:1n − 9; ^+^ Totals include 20:2n − 6 and 22:4n − 6; ^&^ Totals include 18:4n − 3, 20:3n − 3, 21:5n − 3 and 22:5n − 3.

## Data Availability

Data are contained within the article.
